# Urinary volatile organic compound metabolites and COPD among US adults: mixture, interaction and mediation analysis

**DOI:** 10.1186/s12940-024-01086-y

**Published:** 2024-05-03

**Authors:** Ying Wang, Zhaowei Meng, Sen Wei, Xuebing Li, Zheng Su, Yong Jiang, Heng Wu, Hongli Pan, Jing Wang, Qinghua Zhou, Youlin Qiao, Yaguang Fan

**Affiliations:** 1 Department of Radiology, Tianjin Medical University General Hospital, Tianjin, 300052 China; 2 Department of Nuclear Medicine, Tianjin Medical University General Hospital, Tianjin, 300052 China; 3 Department of Lung Cancer Surgery, Tianjin Medical University General Hospital, Tianjin, 300052 China; 4 Tianjin Key Laboratory of Lung Cancer Metastasis and Tumor Microenvironment, Department of Lung Cancer Surgery, Tianjin Lung Cancer Institute, Tianjin Medical University General Hospital, Tianjin, 300052 China; 5 Department of Tobacco Control and Prevention of Respiratory Disease, Center of Respiratory Medicine, China-Japan Friendship Hospital, Beijing, China; 6 National Cancer Center, National Clinical Research Center for Cancer, Cancer Hospital, Chinese Academy of Medical Sciences and Peking Union Medical College, 100021 Beijing, China; 7 Sichuan Lung Cancer Institute, Sichuan Lung Cancer Center, West China Hospital, Sichuan University, Chengdu, China; 8 Center of Global Health, School of Population Medicine and Public Health, Chinese Academy of Medical Sciences and Peking Union Medical College, 100730 Beijing, China

**Keywords:** Volatile organic compounds, Chronic obstructive pulmonary disease, Environmental mixtures, Bayesian weighted quantile sum regression, Quantile-based g-Computation, Bayesian kernel machine regression

## Abstract

**Background:**

Volatile organic compounds (VOCs) encompass hundreds of high production volume chemicals and have been reported to be associated with adverse respiratory outcomes such as chronic obstructive pulmonary disease (COPD). However, research on the combined toxic effects of exposure to various VOCs on COPD is lacking. We aimed to assess the effect of VOC metabolite mixture on COPD risk in a large population sample.

**Methods:**

We assessed the effect of VOC metabolite mixture on COPD risk in 5997 adults from the National Health and Nutrition Examination Survey (NHANES) from 2011 to 2020 (pre-pandemic) using multivariate logistic regression, Bayesian weighted quantile sum regression (BWQS), quantile-based g-Computation method (Qgcomp), and Bayesian kernel machine regression (BKMR). We explored whether these associations were mediated by white blood cell (WBC) count and total bilirubin.

**Results:**

In the logistic regression model, we observed a significantly increased risk of COPD associated with 9 VOC metabolites. Conversely, N-acetyl-S-(benzyl)-L-cysteine (BMA) and N-acetyl-S-(n-propyl)-L-cysteine (BPMA) showed insignificant negative correlations with COPD risk. The overall mixture exposure demonstrated a significant positive relationship with COPD in both the BWQS model (adjusted odds ratio (OR) = 1.30, 95% confidence interval (CI): 1.06, 1.58) and BKMR model, and with marginal significance in the Qgcomp model (adjusted OR = 1.22, 95% CI: 0.98, 1.52). All three models indicated a significant effect of the VOC metabolite mixture on COPD in non-current smokers. WBC count mediated 7.1% of the VOC mixture associated-COPD in non-current smokers.

**Conclusions:**

Our findings provide novel evidence suggesting that VOCs may have adverse associations with COPD in the general population, with N, N- Dimethylformamide and 1,3-Butadiene contributing most. These findings underscore the significance of understanding the potential health risks associated with VOC mixture and emphasize the need for targeted interventions to mitigate the adverse effects on COPD risk.

**Supplementary Information:**

The online version contains supplementary material available at 10.1186/s12940-024-01086-y.

## Background

### Disease burden of Chronic obstructive pulmonary disease (COPD)

COPD is the most common chronic respiratory disease worldwide. It is characterized by persistent respiratory symptoms and airflow limitation. Although age-standardized rates of prevalence, incidence, and mortality have significantly declined, the absolute burden of COPD has continued to increase over the last four decades [1]. High-income North America had the highest age-standardized prevalence of COPD in 2019. Among U.S adults, COPD prevalence ranges from approximately 5 to 20%, contingent on the populations assessed and the disease criteria used [2]. Several factors, including smoking, air pollution, and respiratory infection, were reported to increase the risk of COPD [1].

### Source of Volatile organic compounds (VOCs)and Adverse effects of VOCs on human health

VOCs encompass hundreds of high production volume chemicals such as cleaning products, paints, solvents, personal care products, automotive exhaust, and tobacco smoke [3–5]. These compounds are characterized by their volatility, and are important sources of air pollution and occupational exposure. Although VOCs are essential for many industrial processes and consumer goods, their potential health hazards have raised concerns among researchers, policymakers, and public health professionals. Exposure to certain VOCs may increase the risk for a spectrum of illnesses ranging from mild, such as irritation, to very severe, such as cancer [6, 7]. Human exposure to VOCs principally occurs through different routes, including inhalation, ingestion, and skin contact. However, respiratory inhalation route was significantly associated to the exposure [8]. Accordingly, several VOCs have been associated not only with an increased risk of respiratory diseases, such as asthma and COPD, but also with the exacerbation of symptoms and emergency room visits [9–11]. VOCs have emerged as potential biomarkers for the detection and diagnosis specific diseases, such as COPD and lung cancer [12].

### VOCs and COPD risk

Prior studies on VOCs and respiratory illness have mostly used short-term measurements of VOC air concentrations to characterize exposures, which may not reflect chronic exposure to these compounds [13–15]. Instead, measuring the internal dose based on human specimens, such as blood and urine, is more appropriate for the measurement of VOC exposure because it may better reflect chronic exposures and reduce the intra- and inter-person variations in VOC concentration in the air [16]. Recently, several studies have reported an increased risk of COPD, asthma, or reduced lung function in relation to VOC metabolites [17–21]. Significant associations between metabolites of various VOCs, including 1,3-Butadiene, acrylonitrile, acrylamide, propylene oxide, styrene, benzene, ethylbenzene, o-xylene, styrene, toluene, m-p-xylene and reduced lung function/ emphysema and chronic bronchitis were found in American adults [17, 18, 21–24]. Similarly, metabolites of dimethylformamide, acrolein and 1-bromopropane have been also reported to be associated with pulmonary function decline in the general Chinese population [19, 20, 25]. The association between VOC and COPD might be mediated by oxidative damage or systemic inflammation. A previous study suggested that increased C-reactive protein, a marker of systemic inflammation, significantly mediated 5.39% and 5.87% of the N-ace-S-(N-methlcarbamoyl)-L-cys (AMCC)-associated forced vital capacity and forced expiratory volume in 1 s declines, respectively [19]. Other studies have also demonstrated that the decline in lung function due to single VOC metabolite is partly mediated by oxidative DNA damage, inflammation, and pulmonary epithelial injury [20, 21, 23, 25].

### Novel statistical approaches for VOCs mixture and COPD are needed

Environmental pollutants, including VOCs, are typically in complex mixtures. Traditional regression-based risk assessment methods lead to biased and highly unstable results when confronted with the complex exposure pattern, high correlation, and complicated interactions within environmental mixtures [26–28]. Recently, various statistical approaches have been developed to assess the health effects of environmental chemical mixtures in epidemiological studies, such as weighted quantile sum (WQS) regression and Bayesian Kernel Machine Regression (BKMR) [29–32]. Although the association between VOCs in urine and blood and COPD has been previously evaluated, most of these studies only included limited components of VOC metabolites in their analysis or did not utilized the novel statistical approaches for environmental mixtures [17–20, 22]. Tobacco smoking has been recognized as the most significant risk factor for COPD, serving as the primary non-occupational source of exposure to certain VOCs in the U.S. population [33, 34]. However, no study has analyzed the confounding effect and interaction between smoking and VOC metabolite mixture on the COPD risk. Additionally, the direct and indirect effects of a complex VOC mixture on COPD via a mediator variable have not been previously reported.

### The aims of this study

In this study, we investigated the association between urinary VOC metabolites and COPD risk using several novel statistical methods specific to environmental mixtures and utilized data from the National Health and Nutrition Examination Survey (NHANES) 2011–2020, which offers a representative sample of the adult population in the United States. In addition, for the first time, we performed a stratified analysis to investigate the interaction between smoking and VOC metabolites on COPD risk, and assessed the potential mediating effect of oxidative damage or systemic inflammation on these associations at the mixture level. The above comprehensive analysis provided a less biased and more robust estimation of the single and joint effects of urinary VOC metabolites on COPD risk.

## Methods

### Data source and study population

The data used in this cross-sectional study were derived from the NHANES, which was designed to assess the health and nutritional status of adults and children in the United States. The program utilizes a complex, multistage, probability sampling design to ensure representative results. Various data, including interviews, physical examinations, and laboratory tests, were collected to gather information on a range of health topics. This comprehensive dataset provides a unique opportunity to analyze the associations between multiple environmental exposures and specific health outcomes [35, 36]. Written informed consent was obtained from all participants NHANES. As this study involved a secondary analysis of publicly available data, ethical approval from the institutional review board was not required.

The study included five cycles of NHANES: 2011–2012, 2013–2014,2015–2016, 2017–2020 pre-pandemic. Due to the coronavirus disease 2019 pandemic, data collected from 2019 to March 2020 were combined with data from the NHANES 2017–2018 cycle to form a nationally representative sample of NHANES 2017-March 2020 (pre-pandemic) data [35]. Among the 45,462 participants who completed the questionnaire interview and physical examination, 26,216 provided definite information regarding COPD and urinary VOC metabolite. Subsequently, participants with missing information on body mass index (BMI), family poverty income ratio (PIR), serum cotinine concentration, who were pregnant or younger than 20 years old, or participants with extreme values of VOC metabolites (more than six standard deviations from the mean after log-transformation and centralization of VOCs) were excluded, resulting in a total of 5997 participants included in the analysis. A detailed participant selection flowchart from NHANES 2011–2020 pre-pandemic is illustrated in Figure S1.

### Measurement of urine VOCs

In this study, 18 VOC Metabolites were analyzed. Detailed information on these VOC metabolites, including their full names, parent VOCs, and the proportions at or above the detection limit for the 5997 participants, is presented in Table S1. In the NHANES, spot urine samples were collected from a sub-sample of the participants. The urine specimens were processed, stored, and shipped to the Division of Laboratory Sciences, National Center for Environmental Health, Centers for Disease Control and Prevention, Atlanta GA. The vials were stored under the appropriate freezing conditions (–20 °C) until the assay. The VOC metabolites in human urine were measured performed using ultra-performance liquid chromatography coupled with electrospray tandem mass spectrometry (UPLC-ESI/MSMS). Details on laboratory methods and quality control have been previously described [37]. A imputed fill value was used for analytes with results below the lower limit of detection, an imputed fill value was used. This value is calculated as the lower limit of detection divided by the square root of 2 (LLOD/sqrt [2^2^]). To reduce the effect of urine dilution on the measurements, we used urine creatinine to calibrate the levels of urinary VOC metabolites(ng/g).

### Definition of COPD

Information on COPD was derived from self- and proxy-reported personal interview data using the NHANES MCQ questionnaire section. Specific questions related to COPD were asked for the cycles of 2011–2012, 2013–2014, 2015–2016 and 2017–2018,. Participants were asked whether a doctor or other health professional had ever told them that they had emphysema (MCQ160G, 2011–2018), chronic bronchitis (MCQ160K, 2011–2018), or COPD (MCQ160O, 2013–2016). In the 2019-20 survey cycle, these questions (MCQ160G, MCQ160K and MCQ160O) were replaced by MCQ160P, which asked participants if they had ever been told by a doctor or other health professional that they had chronic obstructive pulmonary disease, or COPD, emphysema, or chronic bronchitis. To ensure compatibility between the 2017-18 data and the 2019-20 data, respondents who answered “Yes” to either MCQ160G, MCQ160K, or MCQ160O were coded as “Yes” to MCQ160P. These respondents were considered COPD patients with COPD.

### Covariates

Characteristics including age, sex, race/ethnicity (non-Hispanic white, non-Hispanic black, Mexican American, and others), education level, PIR, and BMI were obtained from interview questionnaires and examination data. The participants’ smoking status was categorized into three groups: never smokers, former smokers, and current smokers. Never smokers were defined as individuals who smoked < 100 cigarettes in their lifetime. Former smokers were defined as those who had smoked more than 100 cigarettes in their lifetime but had quit smoking at the time of the survey. Current smokers were defined as those who had smoked > 100 cigarettes in their lifetime and were still smoking at the time of the survey. Serum cotinine levels, white blood cell (WBC) count and total bilirubin levels were also measured.

### Statistical analysis

Demographic and personal data, as well as the concentrations of urine VOC metabolite, were compared between non-COPD and COPD patients using the chi-square test for categorical covariates and Kruskal-Wallis test for quantitative data (due to their non-normal distribution). Because the urinary VOC metabolite data exhibited a highly right-skewed distribution (approximating a log-normal distribution), we performed a logarithmic transformation for subsequent analysis. They were then centered to achieve an equal scale, because both BKMR and logistic regression were sensitive to extreme values [38]. We assessed the correlation between the ln-transformed and centralized urinary VOC metabolite data using Pearson correlation analysis.

We used a multivariate logistic regression model to evaluate the effect of a single urinary VOC metabolite on COPD. In the logistic regression model, single urinary VOC metabolite was first included as continuous variable, and then divided into quartiles with the lowest quartile (Q1) serving as the reference group. We used three logistic regression models. Model 0 was not adjusted for any covariates, Model 1 was adjusted for age and sex only, and Model 2 was adjusted age, sex, race, education, BMI, PIR and survey cycle. Furthermore, we examined potential nonlinear dose-response associations between urinary VOC metabolites and COPD using a restricted cubic spline within the logistic regression model. The number of knots was determined using the Akaike information criterion (AIC) for optimal model fit [39].

We then used a novel Bayesian extension of WQS regression, referred to as Bayesian WQS (BWQS), to evaluate the joint effect of urinary VOC metabolite mixture on COPD. In the BWQS, the estimated coefficient mapped to the mixture identifies the association between the overall mixture and the outcome, whereas the estimated coefficients mapped to the weights represent the relative contribution of the corresponding components to the mixture [40]. BWQS regression retains many features of the frequentist WQS regression, but does not require a priori specification of a single direction of effect for the entire mixture or splitting of the original dataset, thereby improving the statistical power, flexibility, and stability of the estimates. Among the 18 VOC metabolites, N-acetyl-S-(2-carbamoyl-2-hydroxyethyl)-L-cysteine (GAMA) and N-acetyl-S-(2-hydroxyethyl)-L-cysteine (HEMA) were excluded from BWQS regression because more than 40% of their results were below the limit of detection. In this study, the chain length in Hamiltonian Monte Carlo algorithm in BWQS model was set to 10,000.

Quantile-based g-Computation method (Qgcomp) was ued to evaluate the overall effect of the VOC metabolite mixture on COPD and the relative importance of the individual constituents in the mixture. As an innovative modeling technique for environmental mixtures, Qgcomp builds on WQS regression by integrating its estimation procedure with g-computation. It estimates the overall mixture effect utilizing the same procedure as WQS, but fits the parameters of a marginal structural model rather than a standard regression [31]. In contrast to WQS, Qgcomp does not enforce the assumption of “directional homogeneity”. This model can estimate the combined effects of the mixtures and elucidate the positive or negative weights of each component. In this study, the number of bootstrap iterations in the Qgcomp model was set to 10,000, the Parameter q was set to 4. Besides, GAMA and HEMA were also excluded.

We used the BKMR model to investigate the combined effect and individual potential nonlinear and interactive effects of urinary VOC metabolites on COPD risk. BKMR integrates Bayesian and statistical learning methods to iteratively regress an exposure–response function using a Gaussian kernel function [29, 41]. The combined effect of the chemical mixture was assessed by calculating the expected outcome variations for the chemicals at specific quantiles compared with those at medians. In the BKMR model, posterior inclusion probabilities (PIPs), an index ranging from 0 (least important) to 1 (most important), provide measures of variable importance for each exposure. The estimated univariate exposure-response function of each VOC metabolite was graphically depicted to examine the potential nonlinearity of the exposure response. Furthermore, bivariate exposure-response curves visualized the interactions between mixture components, with the slopes of the curves of a certain chemical being varied at the 10th, 50th, and 90th percentiles of another chemical (the remaining variables were fixed at the median), which indicated a possible interaction. GAMA and HEMA were also excluded from the BKMR model. The number of iteration was set to 20,000 using the Markov Chain Monte Carlo algorithm.

To explore the potential interaction between urinary VOC metabolites and smoking in COPD, we performed stratified analysis in both logistic regression and mixture analysis by smoking status (non-current and current).

Finally, we explored the potential mediation of the associations by WBC count and total bilirubin levels. We used BKMR-causal mediation analysis (BKMR-CMA) to estimate counterfactually defined estimates of natural direct effects (NDE) and natural indirect effects (NIE) which sum up to the total effect (TE) [42]. In this study, NDEs captured a change in urinary VOC mixtures from the 75th percentile to the 25th percentile while fixing the mediator to the level it would have taken if the exposure was set to the 25th percentile, and considered smoking status (non-current, current smoking) as an effect modifier.

We conducted a series of sensitivity analyses to assess the robustness of our findings. First, as previously described, we applied logistic regression analysis and fitted three models, namely Model 0, Model 1, and Model 2, each with different covariate adjustments. Second, considering the complex multistage sampling design employed in NHANES, we conducted a survey-weighted logistic regression analysis. Third, in both the logistic regression analysis and subsequent mixture analyses, we adjusted for serum cotinine levels instead of the categories of smoking status. All statistical analyses were performed using R software (versions 4.0.2 and 4.2.2; R Core Team), with a significance level set at α = 0.05 for two-sided testing. The key R packages utilized in the statistical analysis and figure production included “survey”, “BWQS”, “qgcomp”, “BKMR”, and “BKMR-CMA”.

## Results

The general characteristics of the 5997 participants are presented in Table 1. The prevalence of COPD was 7.4%. Distributions of age, ethnicity, educational level, BMI, family income and smoking status were significantly different between the COPD and non-COPD participants.

**Table 1:** Participants’ characteristics by COPD in NHANES, 2011–March 2020 pre-pandemic^*^

Characteristics	Total(*n* = 5997)	Non-COPD(*n* = 5552,92.6%)	COPD(*n* = 445,7.4%)	*P* value
Age	49.0 (34.0, 63.0)	48.0 (34.0, 62.0)	61.0 (48.0, 70.0)	< 0.001
< 40	2,013 (33.6%)	1,942 (35.0%)	71 (16.0%)	< 0.001
40–59	2,034 (33.9%)	1,895 (34.1%)	139 (31.2%)	
≥ 60	1,950 (32.5%)	1,715 (30.9%)	235 (52.8%)	
Gender				
Female	2,975 (59.6%)	2,739 (49.3%)	236 (53.0%)	0.146
Male	3,022 (50.4%)	2,813 (50.7%)	209 (47.0%)	
Race				
Mexican American	707 (11.8%)	683 (12.3%)	24 (5.4%)	< 0.001
Other Hispanic	617 (10.3%)	585 (10.5%)	32 (7.2%)	
Non-Hispanic White	2,221 (37.0%)	1,951 (35.1%)	270 (60.7%)	
Non-Hispanic Black	1,468 (24.5%)	1,388 (25.0%)	80 (18.0%)	
Other Race	984 (16.4%)	945 (17.0%)	39 (8.8%)	
Education				
Less than 9th grade	481 (8.0%)	444 (8.0%)	37 (8.3%)	< 0.001
9-11th grade	709 (11.8%)	633 (11.4%)	76 (17.1%)	
High school graduate	1,365 (22.8%)	1,238 (22.3%)	127 (28.5%)	
Some college or AA degree	1,875 (31.3%)	1,735 (31.2%)	140 (31.5%)	
College graduate or above	1,567 (26.1%)	1,502 (27.1%)	65 (14.6%)	
Smoking				
Never	3,394 (56.6%)	3,274 (59.0%)	120 (27.0%)	< 0.001
Former	1,439 (24.0%)	1,282 (23.1%)	157 (35.3%)	
Current	1,164 (19.4%)	996 (17.9%)	168 (37.8%)	
BMI				
<25	1,709 (28.5%)	1,612 (29.0%)	97 (21.8%)	< 0.001
25–30	1,920 (32.0%)	1,805 (32.5%)	115 (25.8%)	
≥30	2,368 (39.5%)	2,135 (38.5%)	233 (52.4%)	
PIR				
< 1.30	1,874 (31.2%)	1,684 (30.3%)	190 (42.7%)	< 0.001
1.30–3.5	2,238 (37.3%)	2,067 (37.2%)	171 (38.4%)	
3.5-	1,885 (31.4%)	1,801 (32.4%)	84 (18.9%)	
Urinary VOC concentrations (ng/g)				
2MHA	27,899.2 (13,677.4, 68,970.6)	27,230.8 (13,518.1, 65,018.4)	45,400.0 (16,160.7, 129,600.0)	< 0.001
34MH	151,141.6 (79,577.5, 444,444.4)	147,129.1 (78,335.9, 422,204.1)	247,887.3 (103,797.5, 829,411.8)	< 0.001
AAMA	51,111.1 (31,764.7, 91,197.2)	50,000.0 (31,406.4, 88,506.1)	68,240.7 (39,387.8, 127,424.7)	< 0.001
AMCC	145,925.9 (81,818.2, 266,666.7)	140,465.9 (79,099.7, 255,043.1)	243,750.0 (128,859.1, 543,209.9)	< 0.001
ATCA	116,666.7 (57,543.9, 217,977.5)	115,669.2 (58,055.6, 216,021.0)	125,535.7 (55,284.6, 260,392.2)	0.097
BMA	6,465.1 (4,020.3, 11,389.8)	6,466.2 (4,041.4, 11,455.9)	6,463.4 (3,777.8, 10,294.1)	0.299
BPMA	4,396.0 (1,760.3, 11,450.0)	4,473.7 (1,808.5, 11,836.2)	3,551.7 (1,416.7, 9,093.0)	< 0.001
CEMA	99,038.5 (62,682.9, 164,565.2)	97,598.9 (61,598.6, 158,594.6)	143,347.6 (84,347.8, 284,033.6)	< 0.001
CYMA	1,645.6 (956.8, 11,409.4)	1,610.6 (947.0, 7,640.2)	3,444.4 (1,180.0, 148,550.7)	< 0.001
DHBMA	314,018.7 (243,396.2, 407,983.2)	308,213.9 (240,528.2, 399,409.9)	388,125.0 (300,970.9, 511,711.7)	< 0.001
GAMA	10,230.8 (6,650.0, 16,625.0)	10,051.8 (6,584.2, 16,219.5)	12,442.4 (8,209.9, 20,781.2)	< 0.001
HEMA	968.2 (553.5, 1,863.3)	961.7 (548.0, 1,815.3)	1,118.0 (601.1, 2,676.5)	< 0.001
HPM2	29,154.9 (18,500.0, 54,137.9)	28,774.9 (18,333.3, 53,274.6)	37,135.9 (21,744.2, 75,111.1)	< 0.001
3HPMA	236,111.1 (147,979.8, 458,823.5)	231,268.7 (146,395.7, 439,120.5)	325,853.7 (177,000.0, 1,143,518.5)	< 0.001
MADA	133,884.3 (95,820.9, 195,312.5)	132,107.8 (94,918.5, 190,909.1)	168,354.4 (113,953.5, 284,210.5)	< 0.001
MHBMA3	4,711.1 (2,923.5, 9,769.6)	4,581.7 (2,865.8, 9,043.8)	7,494.3 (3,895.1, 33,800.0)	< 0.001
PGA	209,375.0 (152,845.5, 292,647.1)	206,153.8 (151,438.1, 284,540.9)	265,600.0 (178,181.8, 408,510.6)	< 0.001
HPMMA	212,857.1 (149,650.3, 410,126.6)	207,998.7 (148,190.0, 384,681.1)	320,000.0 (181,927.7, 1,217,821.8)	< 0.001

* Categorical variables are expressed as number (percentage), while continuous variables are expressed as median and 25th/75th percentiles. Urinary VOC concentrations were correct with urinary creatinine

**Fig. 1:**
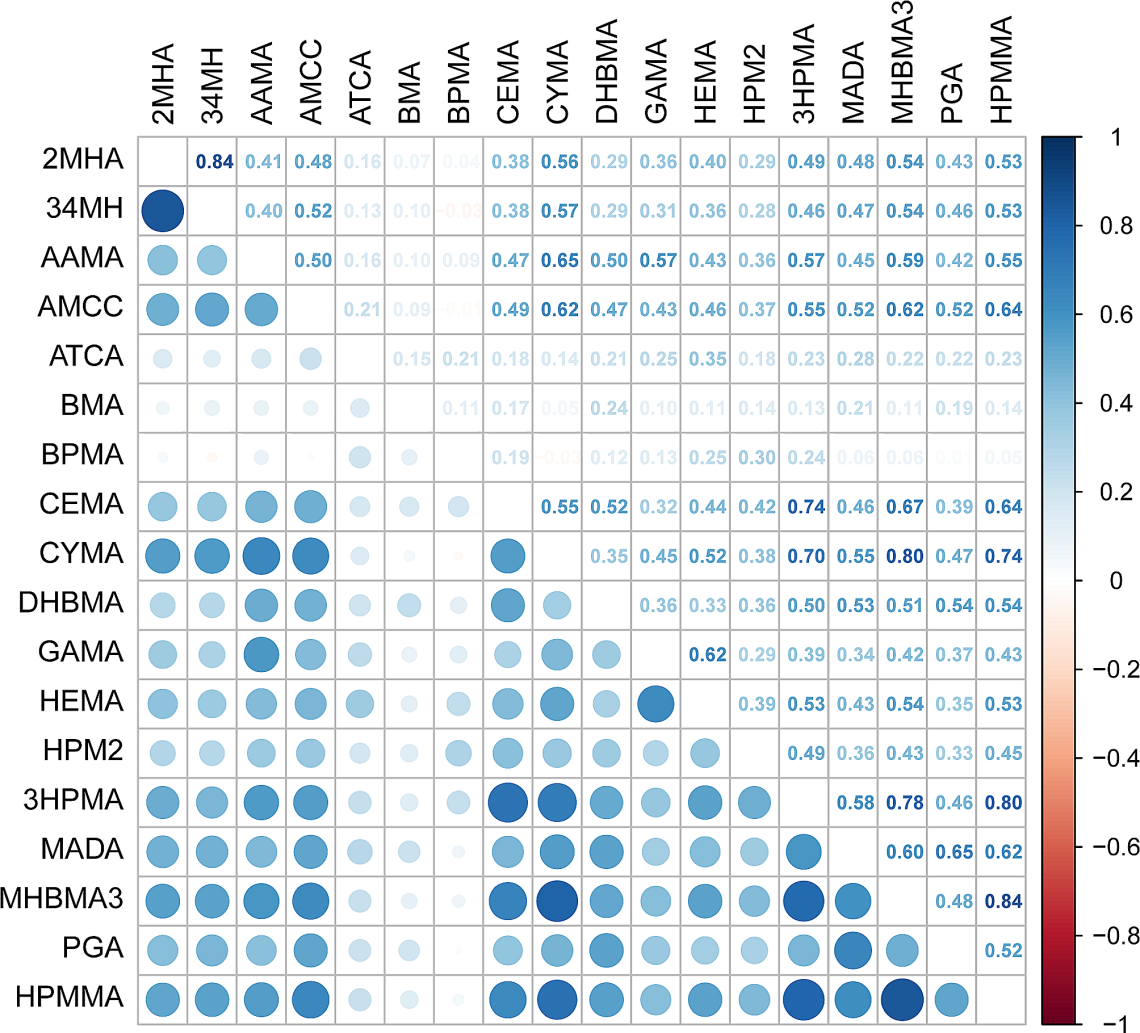
Pearson’s correlation coefficients among 18 urinary VOC metabolites in the population (N=5997), NHANES, USA, 2011-March 2020 pre-pandemic

The detection rates were higher than 75% for 16 VOC metabolites but less than 50% for GAMA and HEMA (Table S1). The creatinine-corrected concentrations of the 18 urinary VOC metabolites are presented in Table 1. COPD patients exhibited significantly higher concentrations of most urinary VOC metabolites, except for 2-amnothiazolne-4-carbxylic acid (ATCA) and N-acetyl-S-(benzyl)-L-cysteine (BMA), whereas the concentration of N-acetyl-S-(n-propyl)-L-cysteine (BPMA) was significantly lower than in those in non-COPD participants. A similar pattern of weighted distributions of general characteristics according to COPD was shown in Table S2. The correlations between the concentrations of 18 VOC metabolites are shown in Fig. 1, ranging from − 0.03 to 0.84. The most highly correlated VOC metabolites were 2MHA and 34MH, MHBM3 and HPMMA, CYMA and MHBMA3, 3HPMA and HPMMA, corresponding of correlation coefficients of 0.84, 0.84, 0.80 and 0.80. This suggests that data analysis with traditional regression method will lead to multicollinearity.

We observed a significant difference in serum cotinine concentrations based on the smoking status, with notably higher levels observed in current smokers (Figure S2). Consequently, we combined never- smokers and former smokers into non-current smokers. Compared with non-current smokers, current smokers possessed higher concentrations of most VOC metabolites, except for BMA and BPMA, which had lower concentrations among current smokers (Figure S3).

**Fig. 2:**
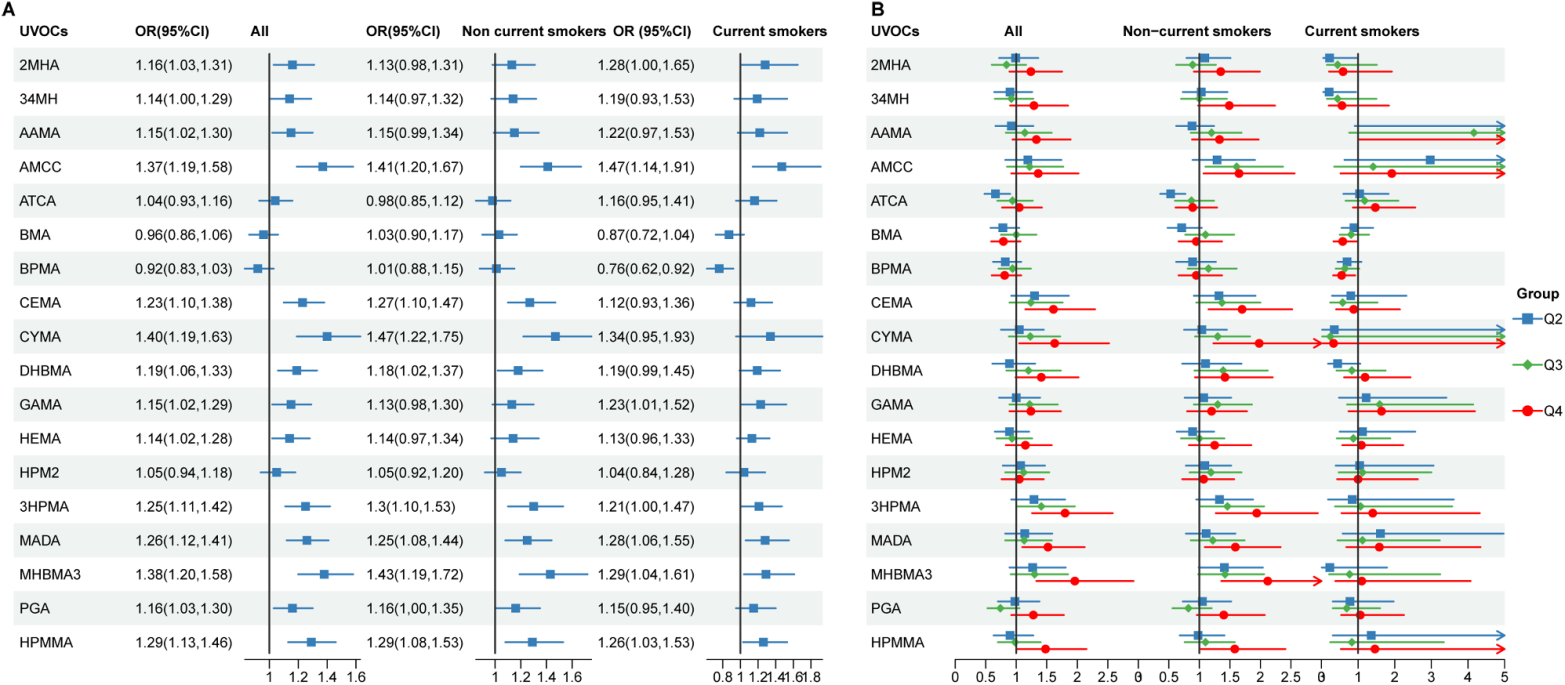
Forest plots for the association of single urinary VOC metabolite with COPD from NHANES 2011–March 2020 pre-pandemic. Adjusted for age, sex, race/ethnicity, education levels, BMI, PIR, survey cycle and smoking status. OR: odds ratio; CI: confidence interval

The associations between urinary VOC metabolites and COPD based on unweighted multivariate logistic regression are presented in Fig. 2A and Table S3. When urinary VOC metabolites were considered as continuous variables, 16 out of 18 urinary VOC metabolites associated with an increased risk of COPD, and significant increase observed for AMCC, N-acetyl-S-(2-carboxyethyl)-L-cysteine (CEMA), N-acetyl-S-(2-cyanoethyl)-L-cysteine (CYMA), Urinary N-Acetyl-S-(3,4-dihydroxybutyl)-L-cysteine (DHBMA), N-acetyl-S- (3-hydroxypropyl)-L-cysteine (3HPMA), Mandelic acid (MADA), N-acetyl-S- (4-hydroxy-2-butenyl)-L-cysteine (MHBMA3), Phenylglyoxylic acid (PGA) and N-acetyl-S-(3-hydroxypropyl-1-methyl)-L-cysteine (HPMMA). Conversely, we found an insignificant decrease in COPD risk with increasing BPMA and BMA levels. Similar patterns were observed when the analyses were repeatedly stratified by smoking status (non-current and current smokers), as shown in Fig. 2A and Table S4. Furthermore, significant non-linear associations between 2-methylhippuric acid (2MHA), 34MH, AMCC, ATCA, MHBMA3, PGA and COPD were observed from the RCS curves (Figure S4).

The 18 urinary VOC metabolites were categorized into four groups based on their quartiles (Fig. 2B and Table S3). After adjusting for age, sex, race, education, BMI, PIR, survey cycle, and smoking status, a significant elevation in COPD risk was observed in the highest quartile (Q4) of CEMA (OR = 1.61, 95% CI: 1.15, 2.29), CYMA (OR = 1.63, 95% CI: 1.05, 2.52), 3HPMA (OR = 1.80, 95% CI: 1.26, 2.58), MADA (OR = 1.52, 95% CI: 1.10, 2.12), MHBMA3 (OR = 1.96, 95% CI: 1.33, 2.92), and HPMMA (OR = 1.48, 95% CI: 1.02, 2.15), when compared to the lowest quartile (Q1). Conversely, although not statistically significant, the BMA and BPMA showed a negative correlation with COPD risk. Similar associations between UVOC metabolites and COPD were also found in non-current smokers. However, these associations fluctuated more among current smokers (Fig. 2B and Table S4).

**Fig. 3:**
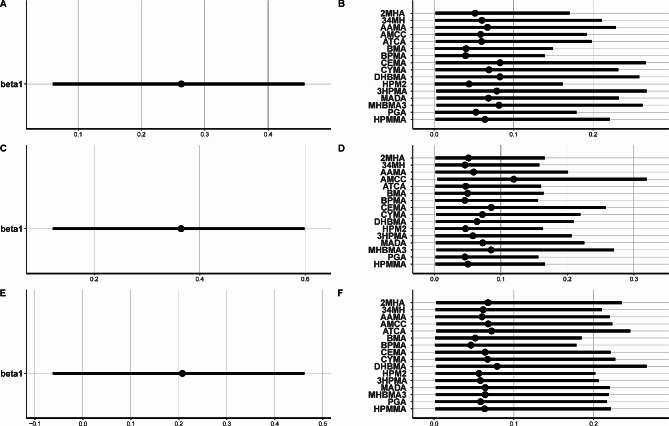
Associations between mixture of 16 urinary VOC metabolites and COPD from BWQS model. Estimates (coefficients) of the association between mixture and COPD in all participants (**A**), non-current smokers (**C**), and current smokers (**E**). Weights with 95% credible intervals for each mixture component in all participants (**B**), non-current smokers (**D**), and current smokers (**F**). Models were adjusted for age, sex, race/ethnicity, education levels, BMI, PIR, survey cycle and smoking status (for all participants)

In BWQS model, we observed a significant association with increased odds of COPD per 1-quartile increase in a mixture of 16 VOC metabolites, with a coefficient of 0.26 (95% CI: 0.06,046), corresponding to an adjusted OR of 1.30 (95% CI: 1.06, 1.58) in all participants (Fig. 3A). Stratified analysis revealed an adjusted OR of 1.44 (95% CI: 1.13, 1.82, Fig. 3C) for COPD per 1-quartile increase in non-current smokers (Fig. 3C). However, this association was not statistically significant in current smokers (OR = 1.23, 95% CI: 0.94, 1.59, Fig. 3E). The contributions of individual VOC metabolites varied based on the smoking status. For all participants, the top two contributors were CEMA and DHBMA. In non-current smokers, the top contributors were AMCC and CEMA, whereas in current smokers, the top contributors were DHBMA and ATCA (Fig. 3B and D, and 3F).

**Fig. 4:**
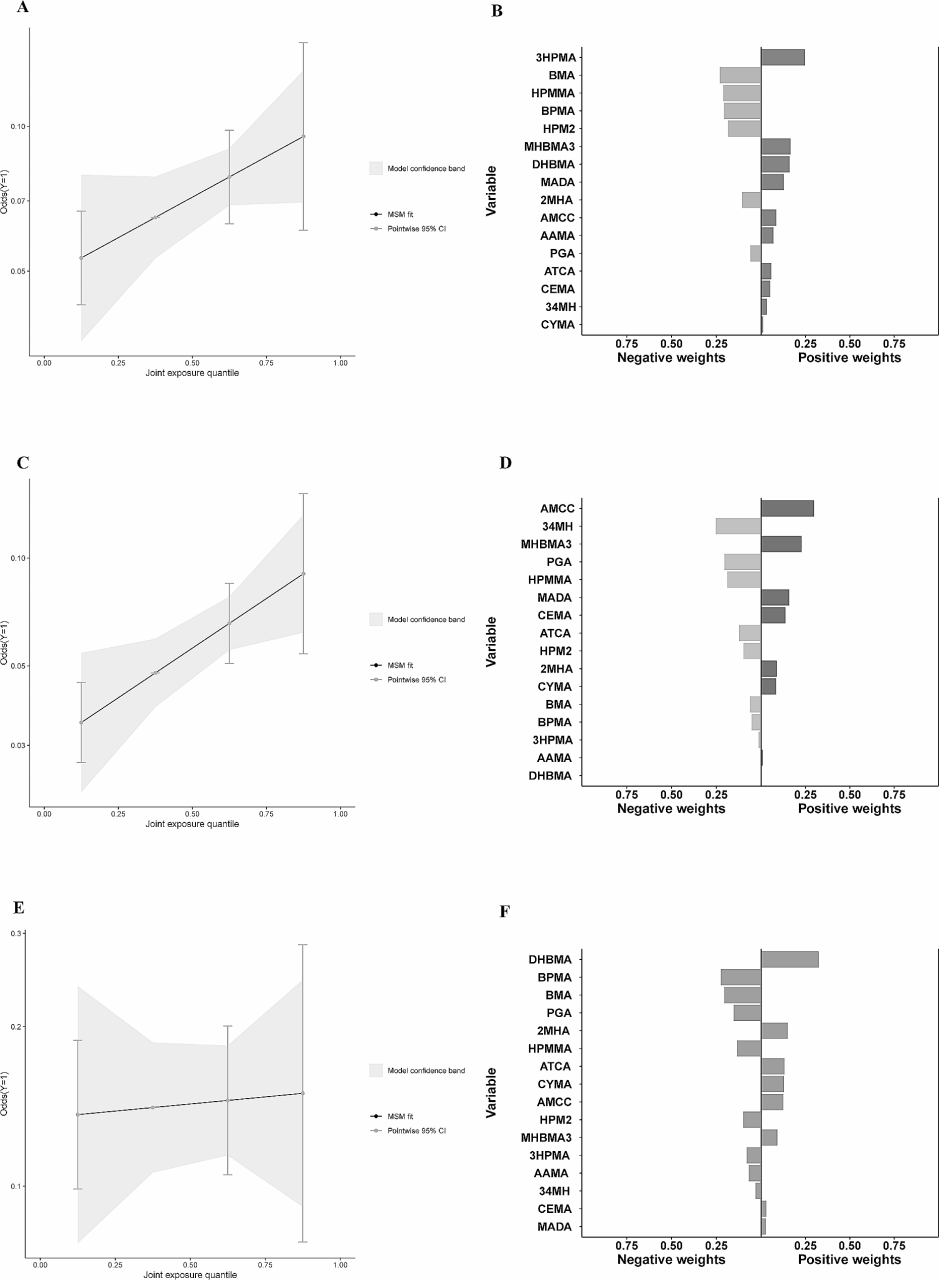
The joint effect (95%CI) and index weights of the mixture of 16 urinary VOC metabolites on COPD risk among all participants (**A**, **B**), non-current smokers (**C**, **D**), and current smokers (**E**, **F**). Qgcomp models were adjusted for age, sex, race/ethnicity, education levels, BMI, PIR, survey cycle and smoking status (for all participants)

The overall effect of the 16 VOC metabolite mixture and the contributors in both the positive and negative directions based on the Qgcomp model are shown in Fig. 4. We observed a marginally significant increase in the risk of COPD for each quartile increase in VOC mixture concentrations, with an adjusted OR of 1.22 (95% CI: 0.98, 1.52) for all participants (Fig. 4A). When stratified by smoking status, this positive association was significant in non-current smokers, with an adjusted OR of 1.37 (95% CI: 1.05–1.77, Fig. 4C). However, no significant relationship between the VOC mixture and COPD was found in current smokers (Fig. 4E). The highest positive weights for all participants, non-current smokers and current smokers were from 3HPMA, AMCC and DHBMA respectively, whereas the highest negative weights were from BMA, 34MH and BPMA (Fig. 4B, D and F).

**Fig. 5:**
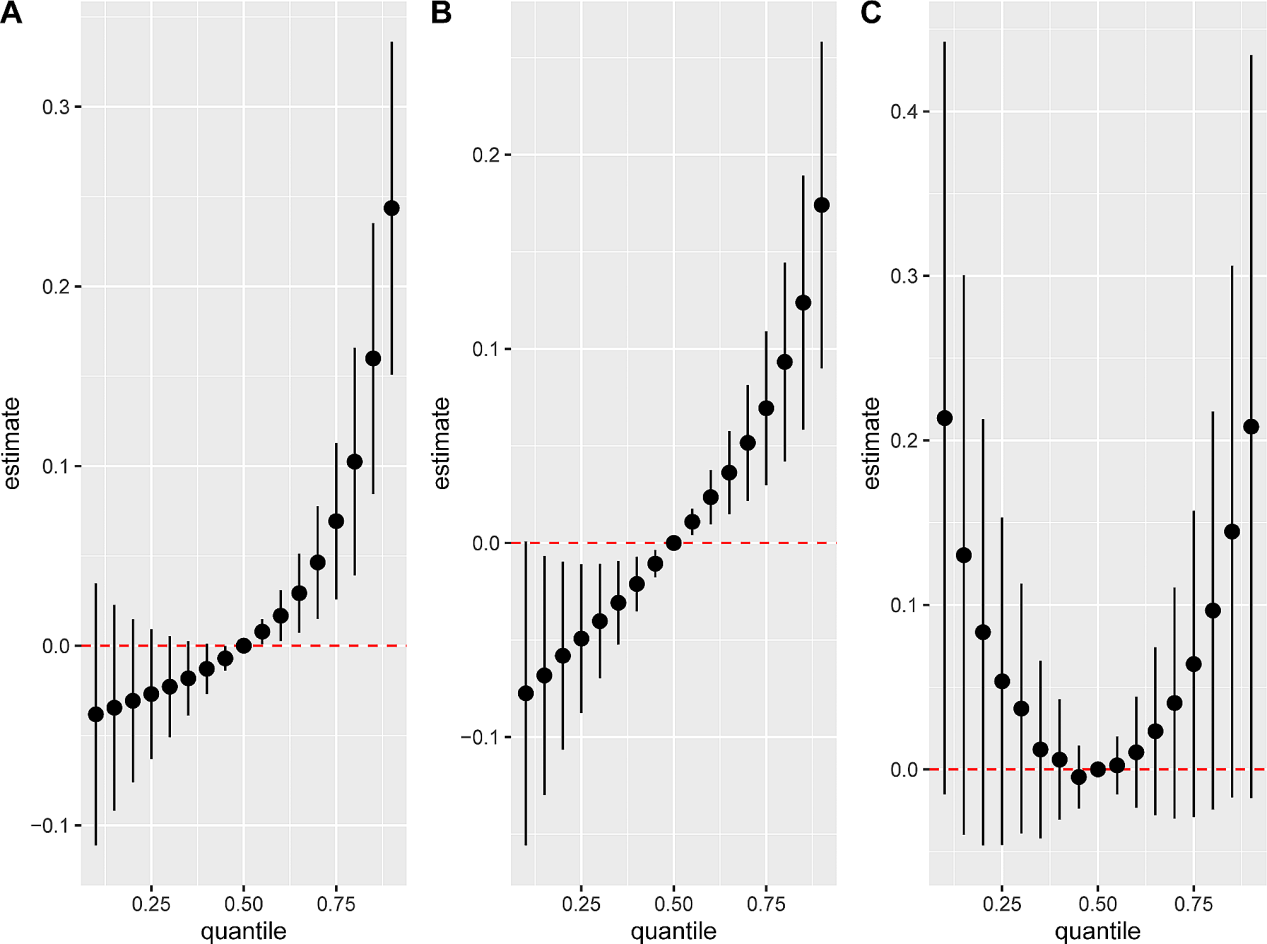
The overall impact of PAHs COPD risk (95%CI) among all participants (**A**), non-current smokers (**B**), and current smokers (**C**). BKMR models were Adjusted for age, sex, race/ethnicity, education levels, BMI, PIR, survey cycle and smoking status (for all participants). BKMR models were adjusted for age, sex, race/ethnicity, education levels, BMI, PIR, survey cycle and smoking status (for all participants)

In all participants, a higher level of VOC metabolite mixture, above the 50th percentile compared to the 50th percentile, was significantly correlated with an increased risk of COPD (Fig. 5A), according to the results from the BKMR model. In the stratified analysis, similar results were observed in non-current smokers (Fig. 5B). However, a U-shaped dose-response curve was observed for the association between the VOC metabolite mixture and COPD risk in current smokers (Fig. 5C).

**Fig. 6:**
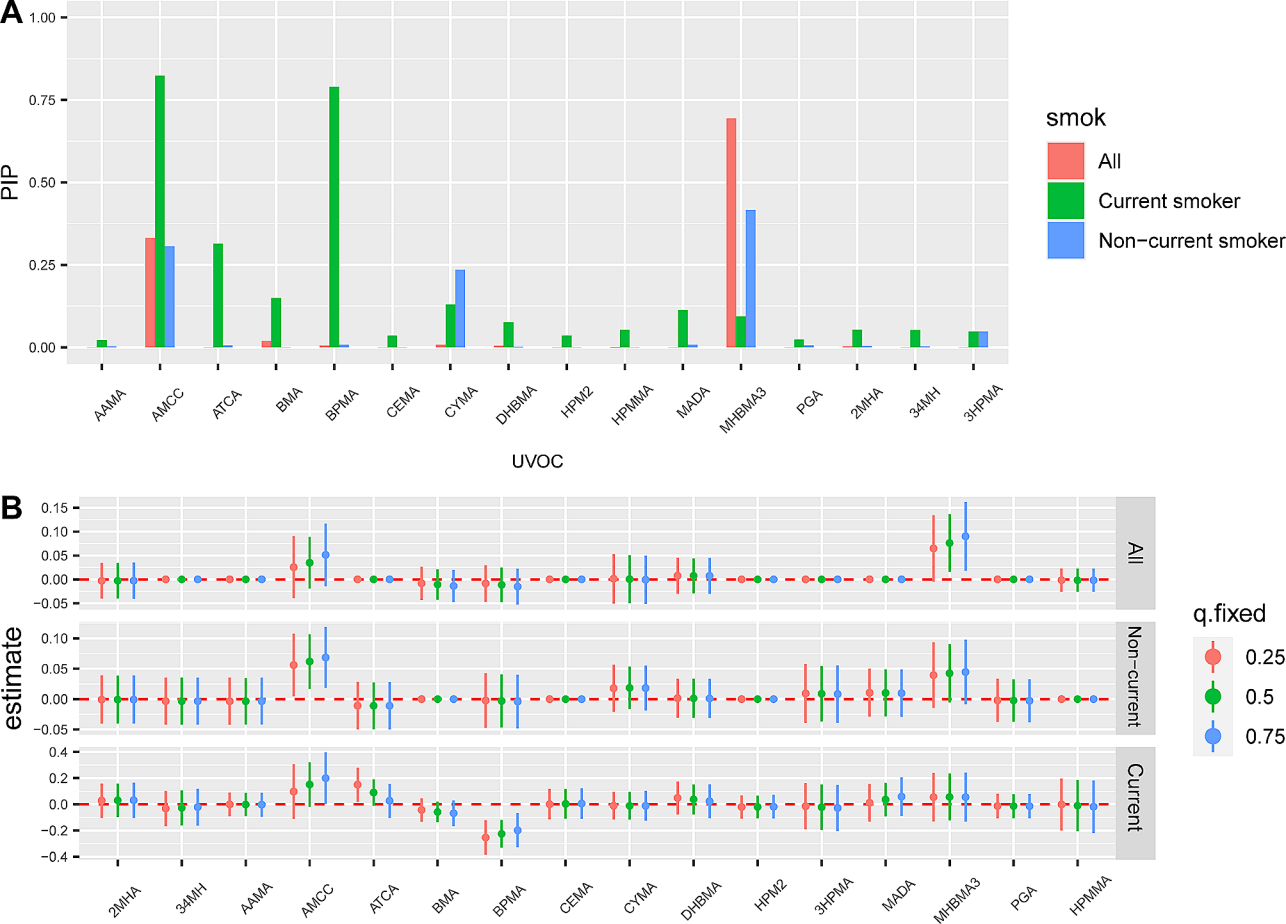
The relative importance (Posterior Inclusion Probabilities) of each urinary VOC metabolite to the COPD risk (**A**). Single effect of urinary VOC metabolite on COPD (**B**), expressed as estimates and 95% CI. These plots compare a latent binary outcome when a single urinary VOC metabolite is at the 75th vs. 25th percentile, when all the other metabolites are fixed at either the 25th, 50th, or 75th percentile. The relative importance (Posterior Inclusion Probabilities) of each urinary VOC metabolite to the COPD risk (**A**). Single effect of urinary VOC metabolite on COPD (**B**), expressed as estimates and 95% CI. These plots compare a latent binary outcome when a single urinary VOC metabolite is at the 75th vs. 25th percentile, when all the other metabolites are fixed at either the 25th, 50th, or 75th percentile. BKMR models were adjusted for age, sex, race/ethnicity, education levels, BMI, PIR, survey cycle and smoking status (for all participants)

The PIP for each urinary VOC metabolite in the mixtures, which determines the VOCs that contribute to the risk of COPD, is shown in Fig. 6A. Among all participants, MHBMA3, AMCC ranked as the top two metabolites with PIPs of 0.90 and 0.53, respectively. In the BKMR model stratified by smoking status, the metabolites with the highest PIPs were MHBMA3 (0.42), AMCC (0.31), and CYMA (0.24) in non-current smokers, whereas in current smokers, the highest PIPs were observed for AMCC (0.82), BPMA (0.79), ATCA (0.31), and BMA (0.15).

The individual effects of each component of the VOC mixture were evaluated by comparing the COPD risk when one component was at the 75th percentile to that when it was at the 25th percentile, while keeping all remaining components fixed at a specific percentile. When the other components fixed at 50th or 75th percentiles, MHBMA3 significantly increased the risk of COPD in all participants. Moreover, significantly increased risk of COPD was observed with an increase in AMCC in non-current smokers, whereas a significantly decreased risk of COPD correlated with an increase in BPMA in current smokers (Fig. 6B).

Figure S5 illustrates the dose-response relationships for each VOC metabolite while setting the other components at their median values. In all participants, AMCC, MHBMA3, and DHBMA demonstrate clear dose-response relationships with COPD risk (Figure S5A). In non-current smokers, significant dose-response relationships were observed for AMCC, MHBMA3, and CYMA (Figure S5B). BPMA levels negatively correlated with COPD among current smokers. Additionally, a U-shaped dose-response relationship between AMCC and COPD was observed in current smokers (Figure S5C).

Furthermore, the bivariate exposure-response functions suggested potential interaction effects of AMCC and ACTA with BPMA on COPD risk in current smokers (Figure S6). The differences in the effect of AMCC on COPD decreased at high levels across the different quartiles of BPMA. No obvious interactions between the two urinary VOC metabolites were observed for all participants and non-current smokers.

Finally, BKMR-CMA was used to estimate the direct and indirect effects of mixtures through the WBC count or total bilirubin level according to smoking status (Table 2). In non-current smokers, WBC count was estimated to account for 7.1% of the association between the urinary VOC mixture and COPD. In current smokers, there was no evidence of an intermediating effect of WBC count on the association between the urinary VOC mixture and COPD. Similarly, we did not observe a mediating effect of total bilirubin on the relationship between the VOC metabolite mixture and COPD.

**Table 2:** Mediation analysis in the association between urinary VOC metabolites and COPD

Mediator	Smoking	Mediation effect	Estimate	95%CI		Proportion
				Lower	Upper	
WBC count	Non-current	Total effect	0.154	-0.144	0.404	1.000
		Natural direct effect	0.144	-0.168	0.472	0.935
		Natural indirect effect	0.011	-0.423	0.337	0.071
	Current	Total effect	0.040	-0.417	0.398	1.000
		Natural direct effect	0.083	-0.631	0.738	2.075
		Natural indirect effect	-0.044	-0.909	0.615	-1.100
Bilirubin	Non-current	Total effect	0.153	-0.092	0.434	1.000
		Natural direct effect	0.163	-0.120	0.502	1.065
		Natural indirect effect	-0.009	-0.337	0.312	-0.059
	Current	Total effect	0.031	-0.377	0.424	1.000
		Natural direct effect	0.035	-0.818	0.749	1.113
		Natural indirect effect	-0.004	-0.525	0.292	-0.129

We performed a weighted logistic regression to evaluate the association between single urinary VOC metabolites and COPD, and the results were generally comparable to those of unweighted logistic regression (Table S5). Additionally, as cotinine is a validated biomarker for cigarette exposure, we repeated the multivariate logistic regression, BWQS and Qgcomp analyses with the adjustment of ln-transformed serum cotinine concentration instead of smoking status, and obtained similar results (Tables S5 and S6).

## Discussion

### The main findings of this study

In this study, we investigated the individual and combined effects of VOC metabolites on COPD in the general population in the United States using multiple statistical strategies. Our findings revealed that of the 18 VOC metabolites examined, 9 showed a positive association with COPD in the multivariate logistic regression analysis. The overall mixture exposure demonstrated a significant positive relationship with COPD in both the BWQS and BKMR models, with a marginal significance in the Qgcomp model. Interestingly, all three models indicated a significant effect of the VOC metabolite mixture on COPD among non-current smokers, but not among current smokers. In contrast, a few other VOC metabolites were negatively or non-linearly correlated with COPD, resulting in a U-shaped dose-response relationship between the overall mixture and COPD. We also found interactions between two VOC metabolites with respect to the risk of COPD. Furthermore, we did not observe a mediating effect of WBC count and total bilirubin on the association between the VOC metabolite mixture and COPD risk.

### Comparison of this study with other studies and the potential explanations

The effects of single VOC metabolites on COPD and pulmonary function have been evaluated in different population. Based on the NHANES data, some studies have reported significant associations between over 10 kinds of VOC metabolites and COPD or reduced lung function [17, 18, 21–24]. Similar results have been observed in Chinese adults. Urinary metabolites of dimethylformamide, acrolein and 1-bromopropane were found to be related to pulmonary function decline in Chinese general population [19, 20, 25]. In this study, we also observed an increased risk of COPD associated with the metabolites of acrylonitrile, xylene, dimethylformamide, acrolein, and ethylene oxide, but no significant association with cyanide, toluene, 1-bromopropane and propylene oxide. However, it should be noted that the effect of a single VOC metabolite on COPD was not entirely consistent across studies. For example, a study based on NHANES demonstrated that toluene exposure may be associated with impaired lung function in smokers, while a significant negative effect of 1-bromopropane on pulmonary function was reported in our study and a Chinese cohort [20]. These discrepancies may be due to differences in study populations, exposure levels, sample types, and confounders.

Most of the aforementioned studies used a generalized linear regression model (multivariate linear or logistic regression) to evaluate the relationship between VOCs and lung function/COPD. However, traditional regression techniques are unable to evaluate the overall effects of high-dimensional and multi-collinear environmental mixtures, and are ineffective in dealing with the nonlinearity and complex interactions between mixture components. In this study, most of the VOC metabolites were found to be moderately or highly correlated. Therefore, we further analyzed the overall effect of urinary VOC metabolite mixture using three novel methods, including BWQS, Qgcomp and BKMR models. The BWQS regression model estimates associations between mixture and outcome based on linear and additive effect assumptions [40]. The Qgcomp model estimates the overall mixture effect using the same procedure as WQS, but estimates the parameters using a marginal structural model rather than a standard regression, which overcomes the assumption of unidirectionality [31]. BKMR can accommodate the nonlinear and non-additive effects of the multivariate exposure in a flexible non-parametric way. Thus, the strengths, limitations, and eventual complementation of these three models will be uncovered through a joint interpretation after evaluating the different aspects. This study demonstrated that both the BWQS and BKMR models had a significant positive impact on the COPD risk from VOC mixture, whereas the association was only marginally significant in the Qgcomp model. Similar results were found in a recent study that demonstrated a significant joint effect of VOC metabolites on emphysema and chronic bronchitis, both independently and in combination [23]. In addition, both studies demonstrated that MHBMA3 and AMCC contributed the most to the joint effect. However, the study did not explore the interaction between smoking and a VOC metabolite mixture on COPD risk.

In this study, BMA and BPMA were not significantly correlated with other VOC metabolites, suggesting that the sources of their parent VOCs were different from those of the other VOCs. In multivariate logistic regression analysis, we found that BMA and BPMA were negatively correlated with COPO, especially in smokers. Moreover, significant nonlinear associations between several other VOC metabolites and COPD were observed in both the multivariate logistic regression and the univariate dose-response curve in the BKMR model, which might account for the U-shaped joint effect of the VOC metabolite mixture on COPD. These results are contrary to those from another study in China, which found a significantly positive relationship between BPMA and reduced lung function [20]. However, this study only investigated of single effect of BPMA using linear mixed model; the influence of other VOC metabolites was not considered. Furthermore, a recent study found no significant contribution of BPMA on Chronic bronchitis or emphysema at mixture level [23]. Thus, further researches are required to evaluate the true effects of BMA, BPMA on the risk of COPD.

The reasons for the negative associations between BMA, BPMA and COPD risk are unknown. However, altering scenarios may contribute to an explanation. First, it is important to note that most VOCs have been reported to have various acute and short-term effects, including respiratory tract irritation [43]. This could potentially lead individuals who are sensitive to these effects to reduce their exposure to VOCs. However, these sensitive populations may also be susceptible to asthma or COPD. In fact, one study found a significant association between asthma and an increased risk of CB, emphysema, and COPD [44]. Second, the increased risk of COPD due to lower levels of certain VOCs metabolites might be due to the prevalence of patients with COPD who have already reduced their exposure due to illness. This highlights an inherent bias in cross-sectional studies, as risk factors and outcome information are obtained simultaneously. In addition, we used a fixed value to replace values that fell below the lower limit of detection. This may also contribute to the U-shaped joint effect of the VOC metabolite mixture and the nonlinear dose-response relationship in COPD.

All three novel models estimated the relative contributions of the individual components of the VOC metabolite mixture to COPD risk, but the results were not entirely consistent. For all participants, the BWQS and BKMR models identified CEMA and AMCC as the most important contributors. In addition, the Qgcomp model highlights 3HPMA as the most positive contributor. This inconsistency may arise from the nonlinear associations observed between several VOC metabolites and COPD risk, particularly for AMCC, which is evident in both the RCS curve and the univariate dose-response cure in the BKMR model. Furthermore, both MHBMA3 and DHBMA, which are metabolites of 1,3-Butadiene, consistently displayed high weights in all three models. Uniquely, the Qgcomp model provided weights for the mixture components in both the positive and negative directions, and the results of this study indicated that BMA, HPMMA and BPMA were the top three negative contributors to COPD. The negative effects of BMA and BPMA in the Qgcomp model aligned with the results of the multivariate logistic regression and the BKMR model. However, the negative effect of HPMMA in the Qgcomp model contradicts the previous findings. These emphasizes the need for diverse statistical methods to estimate the joint effect of mixture, and for the results to be interpreted collectively, considering their respective advantages and limitations.

Tobacco smoking has been recognized as the most significant risk factor for COPD, serving as the primary non-occupational source of exposure to certain VOCs in the U.S. population [33, 34]. Therefore, we performed stratified analyses in non-current smokers and smokers. While we observed a similar dose-response relationship between the urine VOC metabolite mixture and COPD risk, its overall effect was found to be insignificant in current smokers, which might potentially be attributed to the limited sample size and the increased risk of COPD with both higher and lower exposure compared to the median level of the urinary VOC metabolite mixture. Moreover, the effects and relative importance of individual VOC metabolites differed between non-current and current smokers. One possible explanation for these differences is that the concentrations of some VOCs in non-smokers were substantially lower than those in smokers, often falling below the limit of detection [33]. Another contributing factor may be the more complex interactions between a single VOC metabolite (such as AMCC and BPMA in this study) and components within the mixture.

However, the mechanisms underlying the association between VOCs and COPD remain unclear. Oxidative damage and inflammation might be the two main reasons that account for obstructive lung diseases. A previous study suggested that increased C-reactive protein, a marker of systematic inflammation, significantly mediated 5.39% and 5.87% of AMCC-associated forced vital capacity and forced expiratory volume in 1 s declines [19]. Other studies have demonstrated that the decline in lung function due to single VOC metabolites is partly mediated by oxidative DNA damage, inflammation, and pulmonary epithelium injury [20, 25]. It has been reported that the effect of a combination of selected VOC metabolites on chronic bronchitis and emphysema is also mediated by inflammation [23]. However, a mediation analysis of the mediators of the association between VOC metabolite and COPD at the mixture level has not yet been reported. In this study, we used a novel method to evaluate the mediating effects of the WBC count and total bilirubin, which are markers of systemic inflammation and oxidative stress [45, 46]. We found that in non-current smokers, WBC count mediated 7.14% of the VOC mixture associated-COPD, but this mediating effect was not significant. In addition, no mediation effects were found for WBC count in current smokers or total bilirubin in either non-current or current smokers. however, the mechanisms underlying the association between VOCs and COPD require further investigation.

### Strengths and limitations

This study had several strengths. First, it is based on a nationally representative population with a large sample size. Second, we incorporated three novel statistical models, specifically developed for environmental mixtures, to evaluate and visualize the overall effect of VOC metabolite mixture and explore the mediation effect of markers of inflammation and oxidative stress on VOC mixture. Third, the analysis of VOC metabolites in urine offers advantages such as the relatively longer physiological half-life of these metabolites compared to their parent compounds, as well as the specificity of most metabolites [4]. However, this study had some limitations. First, the measured VOC metabolites in this study only reflect recent exposure, whereas COPD is a disease that develops over an extended period, potentially introducing confounding effects due to long-term variation in these metabolites. Additionally, the cross-sectional nature of the data limites our ability to establish a causal relationship between VOC metabolite mixture and COPD risk. Moreover, this study has inherent biases such as information bias. Second, although the NHANES survey’s weighted sampling design helped reduce selection bias with regarding age, sex, and ethnicity, we opted to use unweighted data for our analysis. This decision was based on the fact that these covariates had already been adjusted in the statistical models in this study, thus, an unweighted estimation was recommended [47]. Third, other risk factors for COPD, such as air pollution, occupational exposure, diet and childhood respiratory infections are associated with VOCs [48]. Therefore, although we conducted statistical adjustments, stratified analyses, and sensitivity analyses to control for potential confounders, residual confounding from unmeasured covariates cannot be ruled out. Finally, although the single effect of VOCs on COPD was found in the general population in different regions, the joint effect of VOC metabolites has rarely reported, and both studies were based on NHAHES data. Further studies are warranted to externally validate their joint effect and to explore the contributions of individual components in other population.

## Conclusions

Using novel statistical methods, we observed positive relationships between a mixture of urinary VOC metabolites and the prevalence of COPD in the general population of the United States, particularly among non-current smokers, these associations were not significantly mediated by systematic inflammation and oxidative stress. N, N- Dimethylformamide, 1,3-Butadiene appeared to contribute mostly to the toxic effect of the VOC metabolite mixture on COPD. The overall effect of the mixture and the relative weight of individual mixture components varied depending on the smoking status. This study provides a more realistic and holistic understanding of the health impacts of VOC mixtures on COPD and has significant implications for the prevention and management of COPD in the general population. Additional prospective and mechanistic studies are warranted to explore the causal association between VOCs and the risk of COPD.

### Electronic supplementary material

Below is the link to the electronic supplementary material.

Supplementary Material 1

## Data Availability

The datasets used for these analyses are publicly available in the NHANES repository.

## Abbreviations

VOCs: Volatile organic compounds
COPD: chronic obstructive pulmonary disease
NHANES: National Health and Nutrition Examination Survey
BWQS: Bayesian Weighted Quantile Sum regression
Qgcomp: Quantile-based g-Computation
BKMR: Bayesian kernel machine regression
OR: Odds ratio
CI: Confidence interval
WQS: Weighted quantile sum
WBC: White blood cell
BMI: Body mass index, PIR, family poverty income ratio
LLOD: Lower limit of detection
2MHA: 2-methylhippuric acid
34MH: 3- and 4-Methylhippuric acid
AAMA: N-Ace-S-(2-carbamoylethyl)-L-cys
AMCC: N-ace-S-(N-methlcarbamoyl)-L-cys
ATCA: 2-amnothiazolne-4-carbxylic acid
BMA: N-acetyl-S-(benzyl)-L-cysteine
BPMA: N-acetyl-S-(n-propyl)-L-cysteine
CEMA: N-acetyl-S-(2-carboxyethyl)-L-cysteine
CYMA: N-acetyl-S-(2-cyanoethyl)-L-cysteine
DHBMA: Urinary N-Acetyl-S-(3,4-dihydroxybutyl)-L-cysteine
GAMA: N-acetyl-S-(2-carbamoyl-2-hydroxyethyl)-L-cysteine
HEMA: N-acetyl-S-(2-hydroxyethyl)-L-cysteine
HPM2: N-Acetyl-S-(2-hydroxypropyl)-l-cysteine
3HPMA: N-acetyl-S- (3-hydroxypropyl)-L-cysteine
MADA: Mandelic acid
MHBMA3: N-acetyl-S- (4-hydroxy-2-butenyl)-L-cysteine
PGA: Phenylglyoxylic acid
HPMMA: N-acetyl-S-(3-hydroxypropyl-1-methyl)-L-cysteine
